# Supplementary figures and images for: Designing a multidimensional vulnerability index for supervising dengue cases from 2015 to 2020 in a low/middle-income country: A spatial principal component analysis
Source: PLoS Negl Trop Dis. 2025 Oct 7;19(10):e0013556. doi: 10.1371/journal.pntd.0013556 (PMC12517521; doi:10.1371/journal.pntd.0013556)

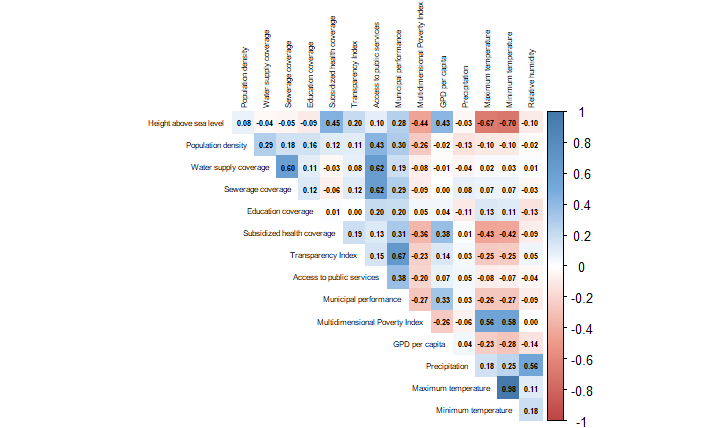

Supplement: S1 Fig — (TIF) [file pntd.0013556.s001.tif]

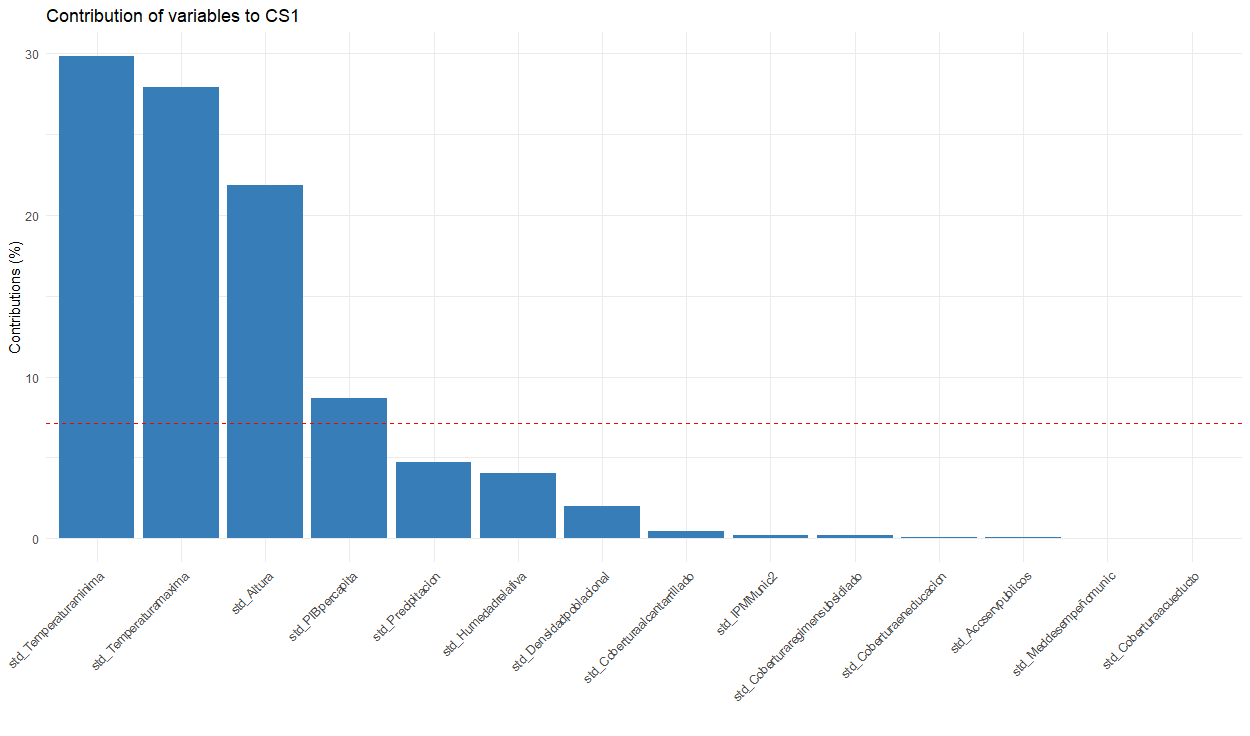

Supplement: S2 Fig — (TIF) [file pntd.0013556.s002.tif]

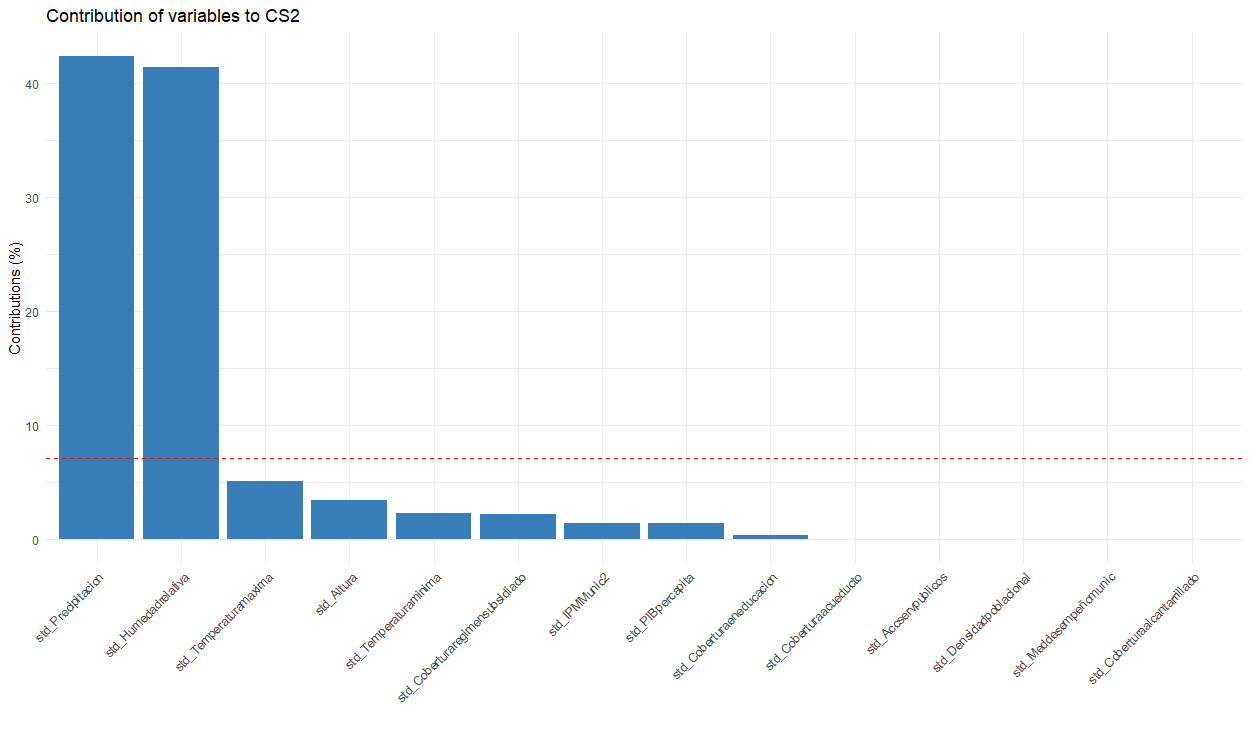

Supplement: S3 Fig — (TIF) [file pntd.0013556.s003.tif]

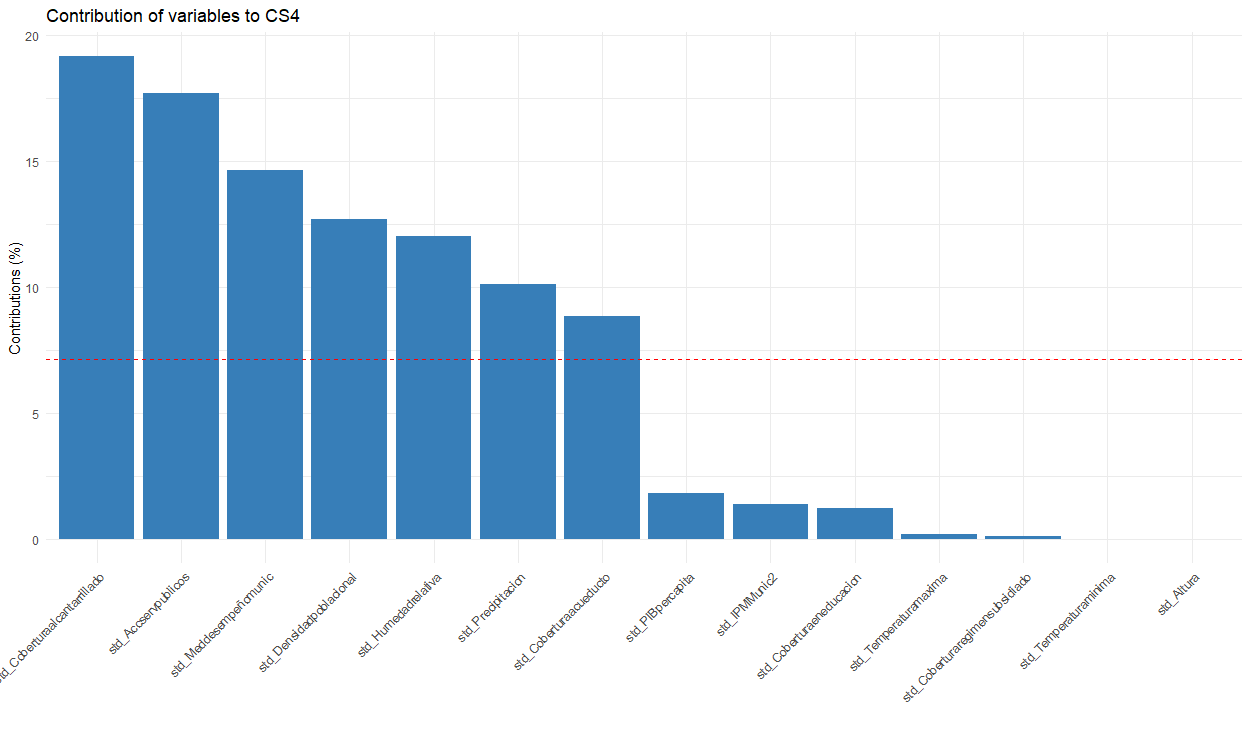

Supplement: S4 Fig — (TIF) [file pntd.0013556.s004.tif]
