# Supplementary material for: Designing a multidimensional vulnerability index for supervising dengue cases from 2015 to 2020 in a low/middle-income country: A spatial principal component analysis
Source: PLoS Negl Trop Dis. 2025 Oct 7;19(10):e0013556. doi: 10.1371/journal.pntd.0013556 (PMC12517521; doi:10.1371/journal.pntd.0013556)
Supplement: Attachment — Submitted filename: Response to reviewers.docx [file pntd.0013556.s006.docx]

Dear editor,

We appreciate all the comments provided by the editors and reviewers to our manuscript. Attached here is the letter that respond to each point raised by the reviewers, the marked copy of the manuscript and the unmarked version of the revised paper.

Reviewers' comments:

**Reviewer #1 comments:**

**Thanks for your comments and the time spent improving the quality of our work. We performed all the suggested changes in the manuscript. Your feedback and thoughtful comments significantly improved our manuscript. All the changes are highlighted in yellow and specified as follows:**

| **Reviewer #1 comments** | **Author’s answer** | **Location of the changes** |
| --- | --- | --- |
| The study design in terms of chosen data is appropriate however the technique requires a major revision. 1) The method is poorly written up with notation not defined, equations formatted poorly and the method not justified. In addition, both PCA and factor analysis are referred to - I wonder which was used. 2) The data is inherently spatial and exhibits spatial autocorrelation as can be seen in the graphics provided, thus a spatial PCA needs to be conducted, not a tradition al PCA. | Thanks for your comment, we appreciate your feedback about our manuscript. We have provided a more specific description of the methodology used in this study. We have clarified the steps and methods used, the notations for each equation, and the additional suggested analyses. About the spatial PCA, we included the specifications of the models Based on these results, the relevance of spatially constrained PCA (spPCA) was evaluated. As the variables exhibited low spatial correlation, a traditional PCA was chosen instead of spPCA. After standardizing the variables PCA allowed us to establish the variables comprising each domain of the index. Moreover a bivariate spatial analysis was also done, the details are described within the methods section. | **Methods section** |
| Reviewer #1: The objective of the paper is clearly stated and the introduction poses this well. Additional literature on existing multidimensional approaches is however missing. | We added additional literature. | **Lines 180-186** |
| Reviewer #1: The results aren't possible to evaluate as the resolution of the graphics is to poor. This will need to be redone before it can be assessed. I do also miss a validation of the index - to just plot the last visualisation does not validate the proposed index. In addition, there is no discussion on the variables removed from the PCA - how does one validate the variables chosen are correct and there isn't something else that needs to be considered? | We added graphs with better quality to improve the visualization. We also added information about the variables removed from the PCA and how that validated the model. | **Figures, methods section: lines** |
| Reviewer #1: The discussion and conclusion make a number of statements that are not shown with evidence in the results.. | We have reviewed and eliminated non-relevant information in both sections. |  |
| Reviewer #1: 1) Throughout: principal component analysis does not get capital letters. 2) ln 65: These statistics are... 3) ln 121: Provide some insight to the reader on the Gini index value. 4) ln 131-132: citations missing 5) ln 139: no capitals 6) lns 141-143: there are many terms here the reader will not understand/know of. Please provide context. 7) ln 147: links needed - add as footnotes 8) ln 149: Some insight from the recommendations should be added to the paper. 9) ln 154: citation missing 10) ln 154: What does a cut off point of > 0.5 mean? 11) lns 176-180: should be in the next section 12) ln 189: How is medium altitude measured?  13) Table 1: why mean for some and median for others? 14) ln 195: Not all readers will know where these areas are? 15) ln 225: no analysis was done, only a visualisation - correct this wording. Add in the actual analysis though to validate the index. 16) ln 236: Where is this significant correlation seen? 17) ln 245-246: language should be fixed 18) ln 248: remove capitals 19) ln 254: where is this spatial analysis that is referred to here? 20) ln 264-265: it is unclear here what you want to say. 21) ln 275-276: there is not evidence provided for this statement. 22) ln 277: this correlation needs to be the spatial autocorrelation in the revision 23) ln 279-280: the results do not show this conclusively. 24) All the references need a lot of formatting and corrections. Links rather as footnotes. Many are not in English - please correct this. | We performed all the suggested changes in the manuscript:   1. We corrected capital letters 2. We corrected misspelling errors 3. We provided info about the Gini index 4. We added the citations 5. We corrected capital letters 6. We added the definitions 7. We added citations that include the links 8. We added this to the discussion section 9. We added the citation 10. We clarified the cutoff point meaning 11. We moved this section 12. We clarified this information, together with the relative humidity, and temperature, were obtained from the DNP databases. 13. We unified all the information, and clarified that mean or median were chosen considering which measure provided more useful information about the data distribution. 14. We clarified this information 15. We clarified this information as suggested, and added the actual analysis. 16. We added this information to the methods section 17. We corrected the language typos. 18. We removed all the unnecessary capital letters. 19. We clarified this information. 20. We clarified this information- 21. Please note that this statement described the findings of this study, not from prior studies. However, we added the reference that supports our findings [32]. 22. We added this clarification. 23. The additional analysis included showed this correlation. |  |
| . Reviewer #1: While interesting, the analysis is flawed and not well explained nor validated. A revision is required. | We added more information about the methodology, and performed additional analysis and descriptions to improve the manuscript. |  |

**Reviewer #2 comments:**

**Thanks for your comments and the time spent improving the quality of our work. We performed all the suggested changes in the manuscript. Your feedback and thoughtful comments significantly improved our manuscript. All the changes are highlighted in yellow and specified as follows:**

| **Reviewer #2 comments** | **Author’s answer** | **Line number of changes** |
| --- | --- | --- |
| Reviewer #2:  1.However, the correlation between the vulnerability index and dengue cases should be explicitly stated to make the hypothesis more testable. 2. However, it is essential to include a section on the dengue surveillance system in the country, detailing how dengue data is collected and the frequency of data collection by various sectors. Additionally, clarifying whether the data was aggregated into annual summaries is necessary.  3. A clear explanation of these terms within the country’s administrative structure is needed to ensure the population is appropriate for the hypothesis being tested.  4. However, further clarification on how the sample size relates to the administrative units and the frequency of data collection would strengthen this assessment.  5. However, the use of epidemiological terminologies should be consistent, and it is important to clarify whether the reported cases are annual averages, incidence rates, or monthly averages. Additionally, the calculation of "Joint spatial distribution" mentioned in Figure 3 should be explained in the methods section. 6. Are there concerns about ethical or regulatory requirements being met? Yes. Please include a map of the country here to highlighting dengue incidence by municipalities/territories. | **1. We clarified this information in the methods and results section and provided wider explanations.**  **2,3,4. We included this information in the methods section (Study design, and dengue cases data)**  **5. We clarified that the reports were annual averages in the methods section of the manuscript.** | **Methods section** |
| Reviewer #2:  Needs improvement. Lines 188 to 189: The statement "The year 2019 had the highest number of reported dengue cases (119,008 cases) with a mean of 4 cases by territory" is unclear. It appears that the country has more territories than municipalities (n=1100), with an average of 108 cases per municipality. Is this figure referring to the monthly average per municipality? Please clarify and revise as needed. Additionally, use appropriate epidemiological terminology (e.g., incidence, rate) and specify whether the data refers to annual average dengue cases or incidence when describing the dengue data. In Table 1, please include the total number of dengue cases for each year and specify whether the averages provided are based on territorial or municipal data. 3. Are the figures (Tables, Images) of sufficient quality for clarity? No. Line 199: The Figure 1 is not clear. Please submit a clear image for review. Line 231: The Figure 2 is not clear. Please submit a clear image for review. Line 231: The Figure 3 is not clear. Please submit a clear image for review. What is meant by the “Joint spatial distribution” mentioned in Figure 3? Please explain how this was calculated in the methods section. Unfortunately, the figures are unclear, making it difficult to provide further comments on this. | **We clarified the information about the number of cases, and clarified that the figure is average per year. And the figure is incidence.**  **Table 1: We included the total number for each year and specify whether the averages provided are based on territorial or municipal data.**  **We improved the quality of the figures.**  **We clarified the information regarding the Spatial Distributions (within the methodology and the results section).** |  |
| Reviewer #2:  Line 236: What is meant by “frequency of dengue cases”? Please use consistent epidemiological terminology throughout the manuscript. Lines 277 to 278: The authors state, "Furthermore, we highlight the correlation between the geographical distribution of the vulnerability index and the incidence of dengue in different regions of the country." This is an important observation that should be supported by the methods and results. Please revise these sections to indicate how this correlation was determined. Additionally, please provide a matrix to identify high-priority municipalities or territories, indicating the recommended operational responses. Please include a section discussing how the vulnerability index can be integrated into dengue prevention and control strategies. Given that this composite index requires intersectoral action, it is crucial to explain how these insights can be translated into actionable steps. Additionally, address how the limitations identified in the data can be mitigated through coordinated efforts across sectors | **We added the word is incidence.**  **We added the information in the methodology and results sections that support this statement.**  **We added additional clarifications regarding the methods section.**  **We added the suggested information to the discussion section. However, as our analysis cannot provide enough information to support causality, we cannot add information about actionable steps, as this may go beyond our scope.** |  |
| #2: The manuscript presents a valuable approach by developing a multidimensional vulnerability index to guide dengue prevention and control strategies. Abstract It is not clearly stated how the vulnerability index correlates with dengue cases. Please include a statement that explains the correlation between the index and dengue incidence across the country. | **We corrected and clarified this information.** |  |
