# Supplementary material for: Designing a multidimensional vulnerability index for supervising dengue cases from 2015 to 2020 in a low/middle-income country: A spatial principal component analysis
Source: PLoS Negl Trop Dis. 2025 Oct 7;19(10):e0013556. doi: 10.1371/journal.pntd.0013556 (PMC12517521; doi:10.1371/journal.pntd.0013556)
Supplement: Attachment — Submitted filename: Response_to_reviewers_auresp_2.docx [file pntd.0013556.s007.docx]

Dear editor,

We appreciate all the comments provided by the editors and reviewers to our manuscript. Attached here is the letter that responds to each point raised by the reviewers, the marked copy of the manuscript and the unmarked version of the revised paper.

**Reviewers' comments:**

**Reviewer #1 comments:**

*Thanks for your comments and the time spent improving the quality of our work. We performed all the suggested changes in the manuscript. Your feedback and thoughtful comments significantly improved our manuscript. All the changes were made using review mode and are specified as follows:*

| **Reviewer #1 comments** | **Author’s answer** | **Location of**  **the changes** |
| --- | --- | --- |
| 1) The response to the reviewers should be properly responded to - not just stated done or edited. Motivate what was done and why. | We appreciate your comments in advance. We have modified the manuscript according to your recommendations, as we performed significant changes to the methodology, and we performed the additional analyses requested by the reviewers, the results and the discussion sections also had significant changes. To clarify your concern, each change will be discussed and specified in detail below. | Methodology, results |
| 2) Spatial PCA is essential - the Moran's I values indicate some very high spatial autocorrelations and the authors have conveniently left out p-values here. The response to this is thus poor. | We appreciate the comment and, based on your observation, we have revised our approach and performed a spatial principal component analysis (spPCA) in the construction of the Multidimensional Vulnerability Index (MVI). To reinforce methodological rigor, we have restructured the methodology section to include:  - The univariate Moran index and associated *p* values for all input variables (see Table 2).  - Justification for the use of MEM (Moran's Eigenvector Maps) in component extraction.  - Step-by-step breakdown of the index construction, incorporating both explained variance and spatial autocorrelation.  - Inclusion of the bivariate Moran's I index between the MVI and dengue incidence in the results.  These revisions improve methodological rigor and consider the spatial structure of the data. | Table 2  Methodology section |
| 3) The authors state they have added literature on other MVI's - but there are not edits in the literature review. Later on there are some citations but that is all focussed on PCA not the design of MVIs. | Thank you for this observation. We included relevant studies on the construction and application of indices in public health and environmental epidemiology. However, we may not have clarified that information enough, so we have now included in the discussion the following references: Cutter et al. (2003), Adger (2006), and additional information about the spatially-informed index approaches. | Line 202 and following at the end of the paragraph |
| 4) Maths equations are poorly formatted. | All mathematical expressions have been reformatted for clarity and consistency, using appropriate equation formatting and notation conventions. | Lines 237 and 241 |
| 5) ln 231: citation missing | We fixed this issue on the manuscript. | Line 246 |
| 6) Factor vs. principal component is still not corrected. | Thank you for your indication. We conducted a careful review of the manuscript and replaced incorrect references to "factors" with "principal components" where appropriate, aligning terminology with PCA theory. |  |
| 7) No where in the methodology is an actual index defined. The GAM is a model - then talk about a model, not an index. But the authors don't do a spatial model for inherently spatial data?? Also not fitted model discussion or information? | This comment was especially helpful and has provided several changes on this section. We have now:  - Added a dedicated subsection on the construction of the index (MVI), including the role of spPCA.  - Clarified that the spatial GAMM is used to validate the theorical association between the constructed index and dengue cases, not to build the index itself.  - Fitted a spatial Generalized Additive Model including a smooth term for longitude and latitude (`s(Lon, Lat)`), which now accounts for spatial clustering.  - Discussed the model structure, significance of terms, and explained variance in the results section. | Line 261 and following at the end of the paragraph |
| 8) references are still poorly formatted | All references have been revised and formatted following the journal’s style guide. We have verified each reference using DOIs and updated the reference management system. | References |
